# Optimal sizing and placement of STATCOM, TCSC and UPFC using a novel hybrid genetic algorithm-improved particle swarm optimization

**DOI:** 10.1016/j.heliyon.2024.e40682

**Published:** 2024-11-23

**Authors:** Urbanus Mwanzia Ngei, Abraham Mutunga Nyete, Peter Musau Moses, Cyrus Wekesa

**Affiliations:** a Department of Electrical, Electronic and Information Engineering, University of Nairobi, Nairobi, Kenya; b Department of Electrical, Electronic and Information Engineering, South Eastern Kenya University, Kitui, Kenya; c School of Engineering, University of Eldoret, Eldoret, Kenya

**Keywords:** FACTS devices, Hybrid GA-IPSO, Multi-objective optimization, Power loss minimization

## Abstract

The increase in global power demand has caused most of today's power networks to become overloaded especially in Sub-Saharan Africa. The increased load demand can be met through expansion of existing generation and transmission system. However, construction of new power infrastructure is limited by financing and technical constraints. Thus, power networks have been left to operate at overload conditions with high power losses and many power quality (PQ) problems. Flexible AC Transmission System (FACTS) devices can improve the power transfer capability of the existing transmission networks without the need of constructing new power infrastructure. In this paper, a multi-objective function comprising of minimization of power loss (PL), voltage deviation (VD) and operational cost (OC) was formulated and solved using a novel algorithm. A novel Genetic Algorithm-Improved Particle Swarm Optimization (GA-IPSO) technique is proposed in this paper for optimization of size and location of FACTS devices. Static Synchronous Compensator (STATCOM), Thyristor Controlled Series Capacitor (TCSC) and Unified Power Flow Controller (UPFC) are the three FACTS devices considered. The proposed technique was validated using IEEE-33 Bus Test System, which is a popular benchmark Radial Distribution System (RDS). The three FACTS devices were optimized separately and also in a combined manner. Under the separate optimization, the size and location of individual FACTS devices were optimized. For combined optimization, the sizes and locations of more than one device were optimized in the same test system. For separate optimization, UPFC produced the best results by reducing the active power losses by 38.44 % and OC from $\${1.59\times 10}^{5}$ to $ ${1.15\times 10}^{5}$. Under the combined optimization, combination of TCSC, STATCOM and UPFC gave better results by achieving active power loss reduction of 56.09 % and reducing OC from $\${1.59\times 10}^{5}$ to $ ${1.03\times 10}^{5}$. Comparison of GA-IPSO technique with other algorithms such as Particle Swarm Optimization (PSO), Genetic Algorithm (GA), Improved Grey Wolf Optimization (IGWO) and Differential Evolution Algorithm (DEA) showed that the proposed hybrid technique was superior and more efficient in solving the FACTS optimization problem.

## Highlights

• Thyristor Controlled Series Capacitor (TCSC), Unified Power Flow Controller (UPFC) and Static Synchronous Compensator (STATCOM) devices were optimized.
• A multi-objective function used to optimize the size and location of FACTS devices.
• GA-IPSO Algorithm used for optimization of sizes and locations of FACTS devices.
• Results showed savings in operating cost and power loss reduction.

## Graphical abstract

**Figure:**
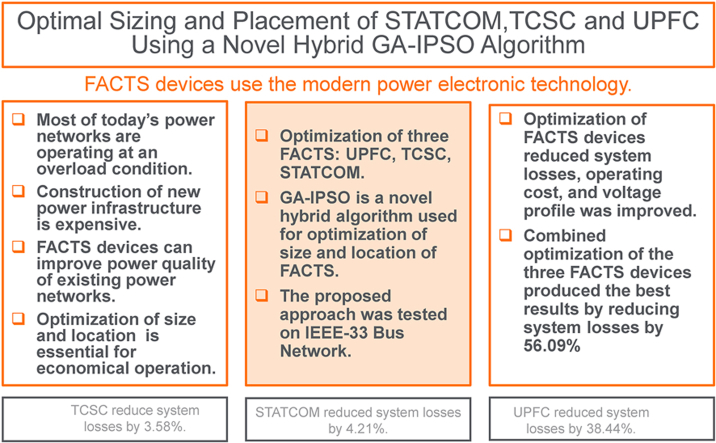

## 1 Introduction

The world has experienced a drastic increase of power demand in the recent years. The witnessed increase in electricity demand has been caused by several factors such as population growth, urbanization and heightened industrial activities [1]. One solution for meeting the increased load demand is through expansion of the existing generation and transmission networks. However, expansion of the power infrastructure is not a feasible solution because of the economic and technical constraints [2]. Furthermore, expansion of existing transmission network is an inappropriate remedy because it can cause congestion, undesirable environmental impacts and health hazards [3]. Another alternative is to increase the transfer capability of power networks by using conventional compensators such as capacitor banks. However, conventional compensators cause harmonics and lead to extreme switching transients. Thus, most of the power networks are left to operate at an overload condition with high power losses, which affects the stability and security of the power system [4,5]. The growth of the global load demand is expected to increase in the future with an approximated demand of 1134.6 GW by the year 2040 [1]. This will increase power quality issues and make future power networks unreliable. Hence, optimal utilization of the existing power networks is the best solution to maintain healthy power networks.

FACTS allows for efficient utilization of the existing transmission and generation capacities with less investment cost [6]. FACTS devices efficiently mitigates voltage instability issue and losses among other power quality problems [7]. These devices use swift power electronics to improve power transfer capability and controllability of power networks [8,9]. Unlike the conventional electromechanical compensators, FACTS devices are compact, which makes them suitable for improving current highly interconnected power systems without adding bulkiness. Since FACTS devices use solid state technology to improve power system performance, these devices do not inject harmonics to the system if controlled by robust controllers and algorithms. FACTS devices can control reactive power under dynamic state; which is suitable for mitigating sudden unexpected changes occasioned by the intermittent Renewable Energy Sources (RES) in today's power networks [10]. Impedance, phase angle and voltage magnitude are fundamental attributes of electrical power systems. The ability of FACTS devices to alter these basic components of power systems allows for enhancement of electrical power networks [11].

The concept of FACTS devices was first introduced in 1999 by Hingorani and Gyugyi [3]. Since then, more advanced and useful FACTS devices have emerged. Different FACTS devices exist and the classification of these devices depend on the method applied to connect them to the power system network. In terms of evolution, FACTS devices can be categorized in two distinct generations. The first generation involve FACTS devices that employ the use of thyristor reactors [3]. Thyristor Controlled Series Capacitor (TCSC) is an example of a FACTS device under this initial generation. Voltage Source Converters (VSC) characterizes FACTS devices under the second generation and a common example is the Static Synchronous Compensator (STATCOM) [5]. Based on the topology used to connect FACTS devices to power network, these devices can be classified as series, shunt and shunt-series [12]. For series FACTS devices, the device is in series with the power network. For a system supplying an inductive load, the voltage drop is $V_{\text{drop}}=\text{IRcos}\varphi +IX_{L}\;\sin\;\varphi$. Connecting a series FACTS device of reactance $X_{C}$ reduces the line reactance to $X_{L}-X_{C}$, which lowers the line's voltage drop. TCSC, Static Series Synchronous Compensators (SSSC) and Thyristor Switched Series Reactor (TSSR) are examples of series FACTS devices. FACTS devices that are connected to power system through parallel connection are referred to as shunt FACTS. This category of FACTS devices operate by consuming or injecting reactive power to the network [13]. Example of shunt FACTS include Static Var Compensator (SVC) and STATCOM. FACTS devices that utilize both series and shunt components to control power system parameters fall under series-shunt FACTS. Thyristor Controlled Phase Shifting Transformer (TCPST) and UPFC are examples of series-shunt FACTS.

Optimization is important in engineering field and all other scientific fields because it allows for efficient use of the available resources. To reap the maximum benefits of using FACTS devices in power networks, optimization of their size and position is inevitable. Failure to optimize the rating of these devices and location where they should be positioned on a power network can increase the cost of running power networks because FACTS devices require a high initial investment cost [14]. Recently, many researchers have directed their attention in finding the best methods that can be applied to place FACTS devices in power systems. Analytic approaches and use of meta-heuristic algorithms are some of the techniques that have been employed to solve the FACTS optimization problem [1]. Meta-heuristic algorithms are robust and flexible and these desirable attributes have made many studies to apply meta-heuristic algorithms to solve the highly complex and highly constrained problem of FACTS devices optimization [5,12,15]. In Ref. [14], GA was applied to optimize TCSC and SVC with an objective to minimize voltage deviation without considering the cost of the FACTS. Adaptive Moth Flame Optimization Algorithm (AMFOA) was used in Ref. [16] to optimize TCSC to improve system power transfer. Again, the investment cost of the FACTS device was ignored in the formulation. Neglecting the cost of the FACTS devices makes it impossible to test their economic feasibility when applied to a particular network.

In [16], STATCOM was optimized using Particle Swarm Optimization (PSO) and the authors did not include the cost in their work. Reference [17] used Particle Swarm Optimization (PSO) for STATCOM optimization on a power network. However, the authors used constant acceleration coefficients limiting the particles' ability to exploit the search space in its entirety. Use of constant acceleration coefficients causes the standard PSO to have reduced exploitation capabilities and can lead to premature convergence. A multi-objective optimization of UPFC was done in Ref. [18] using Lightning Algorithm (LA). The study fell short in testing the same approach on other FACTS devices rather than relying on a single device when testing the method. Different types of FACTS devices control different power system parameters. Thus, combining different FACTS devices can result in better control and performance of power networks. FACTS devices were optimized in Ref. [19] using Artificial Bee Colony Algorithm (ABCA) with two objectives of reducing losses and improving system stability. As other many studies in this research area, authors in Ref. [19] ignored FACTS cost in their design. In Ref. [20], Imperialist Competitive Algorithm (ICA) was used to minimize voltage deviation by optimal placement of TCSC and TCPST. The work did not incorporate the cost for the FACTS installation in the multi-objective function. In Ref. [21], Cuckoo Search Algorithm (CSA) was used to optimize STATCOM without consideration of the FACTS cost. Also, this work did not test the proposed approach in other FACTS devices. In Ref. [22], Firefly Algorithm (FA) was applied to optimize the position of UPFC while CSA was used to optimize it's size. Although this hybrid approach incorporated the cost of UPFC in the objective function, the study considered only one type of FACTS devices. Table 1 is a summary of some past works in the area of FACTS optimization.

**Table 1:** Past works on flexible AC transmission system (FACTS) optimization problem.

Reference	FACTS Devices	Objective(s)	Optimization Approach
[14]	TCSC, SVC	Voltage deviation, line violation	GA
[16]	TCSC	Power transfer capability	AMFOA
[17]	STATCOM	Power loss, cost	PSO
[18]	UPFC	Fuel cost, voltage deviation	LA
[19]	SVC, TCSC, STATCOM	Power losses, system stability	ABCA
[20]	TCSC, TCPST	Voltage deviation	ICA
[21]	STATCOM	Power losses	CSA
[22]	UPFC	Power losses, voltage stability	FA-CSA
[23]	TCSC	Line utilization factor	PSO
[24]	TCSC, SVC	Power losses, voltage deviation	PSO
[25]	STATCOM	Power losses	PSO

A review of the existing literature in FACTS optimization as shown in Table 1 reveal that PSO is a commonly used method when optimizing the location and size of FACTS devices. Features such as less parameters and ease of implementation makes PSO suitable for solving the FACTS optimization problem that is non-linear and involves many constraints [12]. However, conventional PSO suffer from the problem of slow convergence and getting stuck at the local optimum [4].

This paper proposes Genetic Algorithm-Improved Particle Swarm Optimization (GA-IPSO) as a candidate algorithm for finding the optimal size and location of FACTS. The hybrid approach exploits the strengths of the two algorithms while overcoming the weaknesses of the individual optimization techniques. By utilizing the GA-IPSO, the exploitation of the search space will be improved and optimal sizes and positions of different types of FACTS devices will be obtained. A multi-objective function consisting of PL, VD and OC is used. This addresses a common gap where many studies do not consider the FACTS investment cost when optimizing the FACTS devices as indicated in Table 1. In addition, the work is a trailblazer in the field of FACTS optimization because it involves combined optimization of TCSC, STATCOM and UPFC; as opposed to many studies that only consider one type of FACTS device. A combination of different FACTS with unique features can offer better performance of power network and this paper optimizes different combinations of devices from each of the major categories of FACTS devices; shunt (STATCOM), series (TCSC) and series-shunt (UPFC).

This paper's main contributions are.

i) Use of a novel hybrid GA-IPSO algorithm to optimize the location and size of FACTS devices. Hybridization of GA with Improved PSO that uses constriction coefficient restricts the algorithm from getting trapped to local optima.
ii) Combined optimization of different types of FACTS devices to improve performance of power network while ensuring economical operation of the entire power system.
iii) Inclusion of the investment cost of FACTS devices in a multi-objective function of FACTS optimization to ensure economical operation.

## 2 Modelling of FACTS

FACTS devices utilize power electronics technology to improve controllability and the capability of a power grid to deliver power. Through FACTS devices, power transfer parameters of a network can be altered to a achieve a new level of efficiency from an existing network [26]. Power transfer through a transmission system is defined by equation (1).

(1) $$P=\frac{V_{s}V_{r}}{X}*\sin\left(\theta_{s}-\theta_{r}\right)$$

**Fig. 1:**
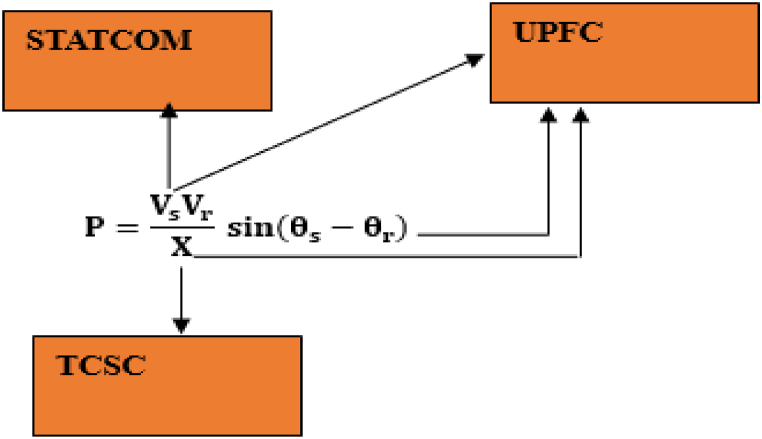
Parameters controlled by different flexible AC transmission system devices.

Where $V_{s}$ is the voltage at the sending side, $V_{r}$ represents the voltage at the receiving side, x is the total reactance of the line, while $\theta_{s}$ and $\theta_{r}$ are phase angles at the sending and receiving side respectively. STATCOM improves power transfer by controlling the voltage, TCSC alters the line reactance to improve the power transmitted; while UPFC can control the voltage, power angle and reactance to adjust the power transmitted [27]. Fig. 1 represents the different parameters controlled by different types of FACTS devices.

The combination of different types of FACTS devices allows for control of all the three parameters, which improves controllability and power transmitted leading to improved performance of the overall electrical network [28]. In this paper, the major classes of FACTS devices are considered: series (TCSC), shunt (STATCOM) and series-shunt (UPFC).

### 2.1 TCSC modelling

TCSC is one of the mostly used series FACTS devices that adds either inductive or capacitive reactance to the transmission line. This device has a thyristor-controlled reactor shunted with a fixed capacitor [29]. By changing the firing angle α of the thyristor, the compensator's reactance can be varied, which alters the overall impedance of the transmission line to improve power transfer. The relationship between the reactance of a TCSC and the firing angle is represented in equation (2).

(2) $$X_{\text{TCSC}}=\frac{X_{C}X_{L}\left(\alpha \right)}{X_{L}\left(\alpha \right)-X_{C}}$$

**Fig. 2:**
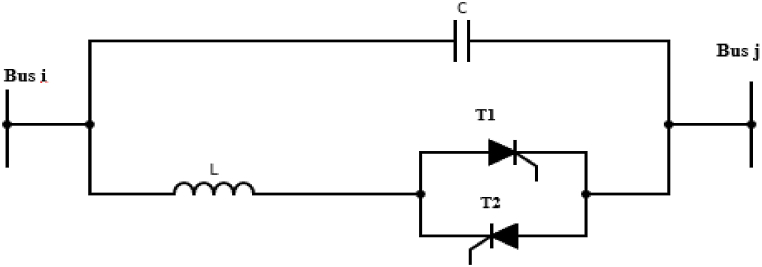
Schematic diagram of thyristor controlled series capacitor (TCSC).

Where $X_{C}$ is the capacitive reactance of the fixed capacitor and $X_{L}\left(\alpha \right)$ is inductive reactance of the thyristor-controlled reactor. The value of $X_{L}\left(\alpha \right)$ is changed by changing the firing angle, and this alters the reactance of TCSC; which is abbreviated as $X_{\text{TCSC}}$. Fig. 2 is a schematic diagram of a TCSC connected between Bus i and Bus j.

**Fig. 3:**
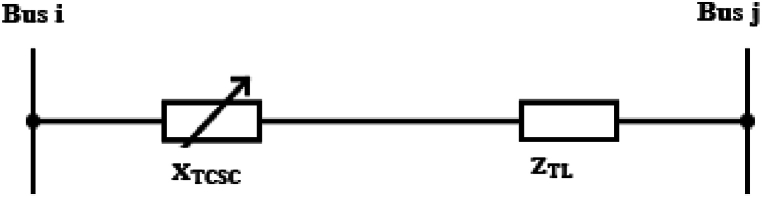
Equivalent Circuit of thyristor controlled series capacitor (TCSC).

TCSC can be modelled as a variable reactance that is connected in series with the impedance of the transmission line as shown in Fig. 3.

The inclusion of TCSC changes the line's reactance as described in equations (3), (4).

(3) $$Z_{\text{TL}}=R_{\text{ij}}+jX_{\text{ij}}$$

(4) $$X_{N}=X_{\text{ij}}-X_{\text{TCSC}}$$

Further simplification of equation (4) yields equations (5), (6) where the ratio of the TCSC reactance to the line reactance before compensation determines the rate of compensation.

(5) $$X_{N}=\left(1-\tau \right)X_{\text{ij}}$$

(6) $$\tau =\frac{X_{\text{TCSC}}}{X_{\text{ij}}}$$

Where $Z_{\text{TL}}$ is the impedance of the transmission line, $R_{\text{ij}}$ and $X_{\text{ij}}$ are the line's resistance and reactance respectively between buses i and j, $X_{\text{TCSC}}$ is the reactance of TCSC, $X_{N}$ is the line's new reactance after introduction of the FACTS device and τ is the rate of compensation. Changing the lines reactance alters the conductance and the susceptance of the line as described in equations (7), (8) respectively.

(7) $$G_{\text{ij}}=\frac{R_{\text{ij}}}{R_{\text{ij}}^{2}+{\;X}_{N}^{2}}$$

(8) $$B_{\text{ij}}=\frac{R_{\text{ij}}}{R_{\text{ij}}^{2}+{\;X}_{N}^{2}}$$

The power flow between bus i and j after incorporating TCSC is describe by equations (9), (10), (11), (12) [30].

(9) $$P_{\text{ij}}={\left|V_{i}\right|}^{2}G_{\text{ij}}-|V_{i}\|V_{j}|\left(G_{\text{ij}}\;\cos\;\delta_{\text{ij}}+B_{\text{ij}}\;\sin\;\delta_{\text{ij}}\right)$$

(10) $$Q_{\text{ij}}=-{\left|V_{i}\right|}^{2}B_{\text{ij}}-|V_{i}\|V_{j}|\left(G_{\text{ij}}\;\sin\;\delta_{\text{ij}}-B_{\text{ij}}\;\cos\;\delta_{\text{ij}}\right)$$

(11) $$P_{\text{ji}}={\left|V_{j}\right|}^{2}G_{\text{ij}}-|V_{i}\|V_{j}|\left(G_{\text{ij}}\;\cos\;\delta_{\text{ij}}-B_{\text{ij}}\;\sin\;\delta_{\text{ij}}\right)$$

(12) $$Q_{\text{ji}}=-{\left|V_{j}\right|}^{2}B_{\text{ij}}-|V_{i}\|V_{j}|\left(G_{\text{ij}}\;\sin\;\delta_{\text{ij}}+B_{\text{ij}}\;\cos\;\delta_{\text{ij}}\right)$$

Where $\delta_{\text{ij}}$ is the voltage phase angle difference between bus i and bus j.

### 2.2 STATCOM modelling

**Fig. 4:**
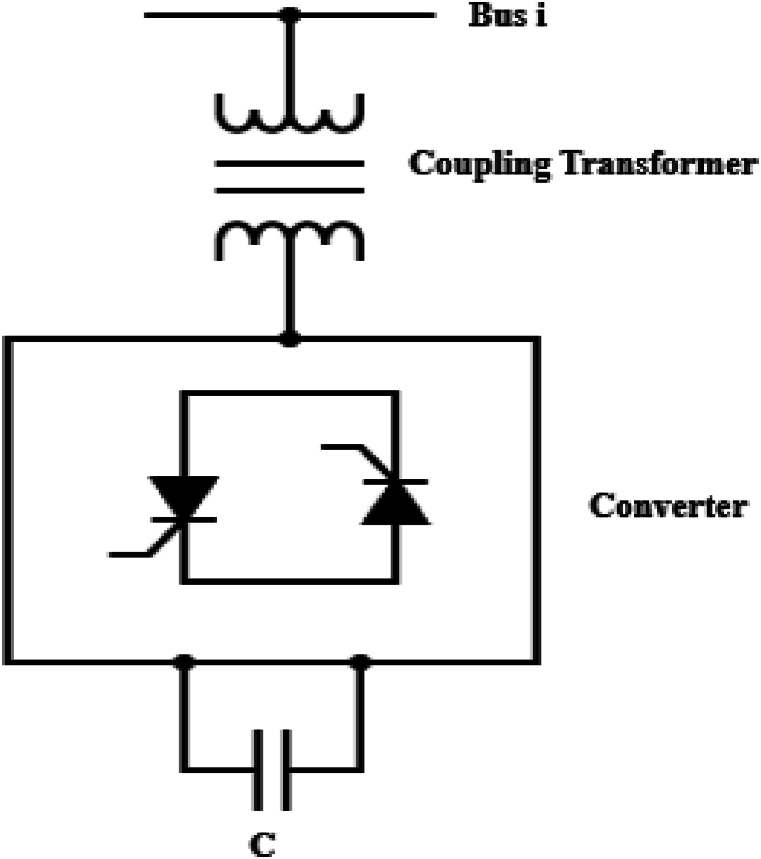
Schematic diagram of static Synchronous compensator (STATCOM).

STATCOM is a shunt-connected FACTS device that consists of a Voltage Source Converter (VSC) and a coupling transformer [31]. Fig. 4 represents a STATCOM connected in parallel with bus i.

**Fig. 5:**
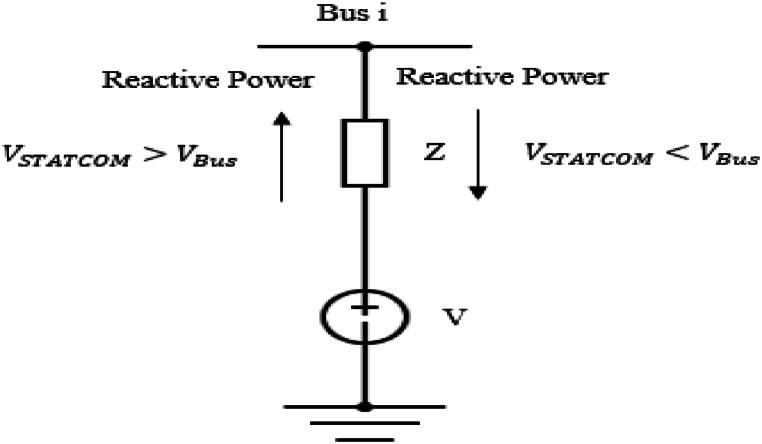
Equivalent Circuit of static Synchronous compensator (STATCOM).

STATCOM is modelled as a voltage source that is in series with the impedance of the coupling transformer. This model of the STATCOM is represented in Fig. 5.

Through the VSC, DC input voltage is converted to AC output voltage of controllable amplitude, phase and frequency. STATCOM works by delivering or absorbing reactive power from the system [32]. Under circumstances when the voltage amplitude of the system is smaller than that of the output of VSC ($V_{STATCOM}>V_{Bus}$), reactive power is supplied to the system by STATCOM as indicated in Fig. 5. Contrary, when the voltage amplitude of the system is bigger than that of the VSC output ($V_{STATCOM}<V_{Bus})$, the STATCOM absorbs reactive power from the system [33]. Under rapid changes of the load reactive power, STATCOM can provide fast response to adjust the system power to the nominal values. Through reactive power compensation, STATCOM helps in reducing power system losses and it improves the system's voltage profile leading to system stability. Thus, STATCOM controls the reactive power of the system and the power flow equations are represented in equations (13), (14), [34].

(13) $$Q_{i}=Q_{S}+\sum_{j=1}^{N}\left|V_{i}\right|\left|V_{j}\right||Y_{\text{ij}}|\sin\left(\theta_{\text{ij}}-\delta_{\text{ij}}\right)$$

(14) $$Q_{s}=B_{s}\left|{V_{i}|}^{2}-\left|V_{i}\right|\right|V_{s}\|Y_{s}|\sin\left(\theta_{\text{is}}-\delta_{s}\right)$$

Where $V_{i}<\theta_{i}$ represents the voltage and phase at bus i, $V_{s}<\theta_{s}$ is the supplied shunt voltage and phase by the STATCOM, $Q_{i}$ is the reactive power at bus i, $Q_{s}$ represents the reactive power of the STATCOM, $Y_{s}$ is the susceptance of the STATCOM, $B_{s}$ is the admittance of the STATCOM, $Y_{\text{ij}}$ represents the line admittance between nodes i and j, while N is the total number of buses connected to bus i.

### 2.3 UPFC modelling

**Fig. 6:**
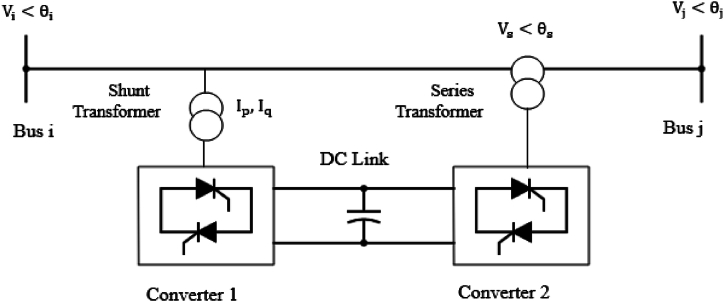
Schematic diagram of unified power flow controller (UPFC).

UPFC is a multipurpose device that can control the impedance, phase angle and voltage magnitude of a power system network. The capability to control all the three power system parameters sets UPFC as a unique and multifunctional compensator that is useful in today's power networks. As shown in Fig. 6, UPFC consists of two converters interconnected through a DC link which allows for bidirectional flow of power. One converter is connected in parallel with the transmission line through a coupling transformer while the other converter is connected in series with the system through a coupling transformer. The parallel converter controls reactive power while the series converter injects voltage with adjustable phase angle and magnitude to the transmission line. Some typical applications of UPFC include voltage enhancement and improvement of both system security and stability.

**Fig. 7:**
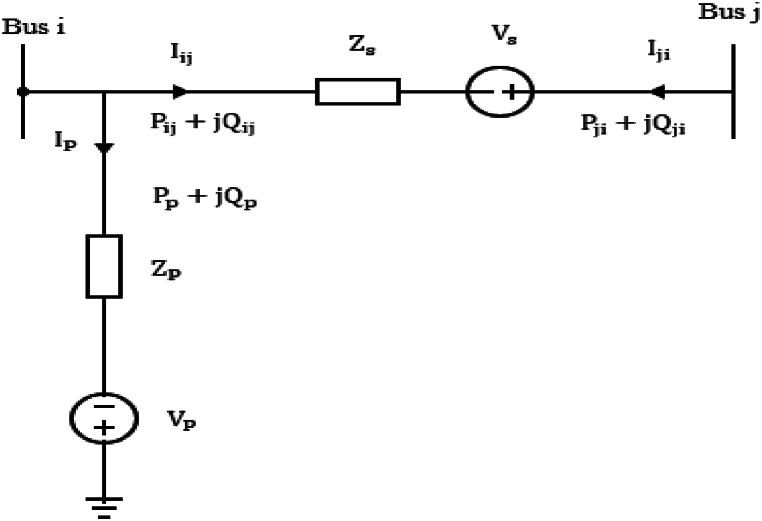
Equivalent Circuit of unified power flow controller (UPFC).

UPFC is modelled using a simple representation of two voltage sources. The first voltage source is connected in series with the impedance of the coupling transformer and in series with the transmission line. The other voltage source is in series with the impedance of the second coupling transformer and shunted with the power network. This model of the UPFC is represented in Fig. 7.

UPFC controls both real and reactive power and the power flow equations between buses i and j after connecting UPFC are presented in equations (15), (16), (17), (18).

(15) $$P_{i}+{V_{s}}^{2}G_{\text{ij}}+2V_{i}V_{s}G_{\text{ij}}\;\cos\left(\theta_{s}-\theta_{i}\right)-V_{j}V_{s}\left[G_{\text{ij}}\;\cos\left(\theta_{s}-\theta_{j}\right)-B_{\text{ij}}\;\sin\left(\theta_{s}-\theta_{j}\right)\right]=0$$

(16) $$Q_{i}+V_{i}I_{q}-V_{i}V_{s}\left[G_{\text{ij}}\;\sin\left(\theta_{s}-\theta_{i}\right)-B_{\text{ij}}\;\cos\left(\theta_{s}-\theta_{i}\right)\right]=0$$

(17) $$P_{j}-V_{j}V_{s}\left[G_{\text{ij}}\;\sin\left(\theta_{s}-\theta_{j}\right)-B_{\text{ij}}\;\cos\left(\theta_{s}-\theta_{j}\right)\right]=0$$

(18) $$Q_{j}-V_{j}V_{s}\left[G_{\text{ij}}\;\sin\left(\theta_{s}-\theta_{j}\right)-B_{\text{ij}}\;\cos\left(\theta_{s}-\theta_{j}\right)\right]=0$$

## 3 Problem formulation

FACTS devices are used for improvement of power system by achieving different objectives. In this paper, minimization of power losses (PL), voltage deviation (VD) and operating cost (OC) are the core objective functions under which optimal allocation of FACTS is obtained while satisfying system constraints. The fitness function is obtained by combining the three objective functions using weighting method to form a multi-objective function for optimization.

### 3.1 Minimization of PL

Real power losses for a power network can be expressed as [5]:

(19) $$\mathrm{Min}\;{\text{OF}}_{1}=\sum_{k=1}^{n}G_{k}\left[V_{i}^{2}+V_{j}^{2}-2V_{i}V_{j}\;\cos\;\left(\delta_{ij}\right)\right]$$

Where, $G_{k}$ is the kth line conductance, n is the number of lines, $V_{i}$ and $V_{j}$ are voltages at bus i and bus j, while $\delta_{ij}$ is the voltage phase difference of buses i and j.

### 3.2 Minimization of VD

Minimizing voltage deviation improves the system's voltage profile while keeping bus voltages within the normal limits. The function for the reduction of bus voltage deviations is represented as:

(20) $$\mathrm{Min}\;{\text{OF}}_{2}=\sum_{i=1}^{n}{|V_{i}-1|}^{2}$$

Where, $V_{i}$ denotes the voltage magnitude at bus i and n is sum of busses.

### 3.3 Minimization of OC

The operating cost is divided in two parts in this paper. There is cost that occurs because of power losses ($C_{\text{LOSSES}}$) and the other cost is as a result of FACTS devices investment ($C_{\text{FACTS}}$) [5]. Under this objective function, the cost due to power loss will be minimized by reduction of real power loss and the investment cost of FACTS devices will be minimized. The objective function to reduce operating cost is represented as:

(21) $$\mathrm{Min}\;{\text{OF}}_{3}=C_{\text{LOSSES}}+C_{\text{FACTS}}$$

(22) $$C_{LOSSES}=Active\;Power\;Loss\times 24\times 365\times 0.09$$

Where, 24 is the total number of hours in a day, 365 is the annual number of days and 0.09 is the global average cost of power in $/kWh [5].

The investment cost of FACTS devices ($C_{\text{FACTS}}$) vary from one device to another. In this paper, the investment costs of the three considered FACTS devices can be expressed as [5,35,36]:

(23) $$C_{\text{TCSC}}=0.0015S^{2}-0.7130S+153.75$$

(24) $$C_{\text{STATCOM}}=0.0003S^{2}-0.3051S+127.38$$

(25) $$C_{\text{UPFC}}=0.0003S^{2}-0.2691S+188.22$$

(26) $$C_{\text{FACTS}}=C_{\text{TCSC}}+C_{\text{STATCOM}}+C_{\text{UPFC}}$$

Where, $C_{\text{TCSC}}$, $C_{\text{STATCOM}}$, $C_{\text{UPFC}}$ are the investment costs of the three FACTS devices in $/KVAr and S is the MVAr range of the FACTS devices.

### 3.4 Multi-objective function

The combined multi-objective function after combining the three objective functions is expressed as:

(27) $$\mathrm{Min}\;F=\omega_{1}{\text{OF}}_{1}+\omega_{2}{\text{OF}}_{2}+\omega_{3}{\text{OF}}_{3}$$

Where $\omega_{1}$, $\omega_{2}$ and $\omega_{3}$ represent the weights to determine the contribution for every objective function in the overall fitness function. The sum of the three weights adds up to 1. In this paper, a trial-and-error approach is used to find the appropriate weighting factors that provide the best balance among the three objective functions.

### 3.5 Constraints

The constraints applied to the optimization problem are listed below:

Power Balance Constraints

(28) $$P_{G\left(i\right)}-P_{D\left(i\right)}=P_{\text{Loss}\left(i\right)}$$

(29) $$Q_{G\left(i\right)}-Q_{D\left(i\right)}=Q_{\text{Loss}\left(i\right)}$$

Where $P_{G\left(i\right)}$ and $P_{D\left(i\right)}$ symbolize real power that is generated and demanded at the ith bus while $Q_{G\left(i\right)}$ and $Q_{D\left(i\right)}$ refer to reactive power generated and demanded at bus i respectively.

### 3.6 Voltage limit constraint

The expression to limit the bus voltages within allowable limits is represented as:

(30) $$V_{i\;\min}\leq V_{i}\leq V_{i\;\max}$$

The value for $V_{i\;\min}=0.90\;p.u$ and $V_{i\;\max}=1.05\;p.u$ [37].

Where $V_{i\;\min}$ is the minimum bus voltage and $V_{i\;\max}$ is the maximum bus voltage.

### 3.7 Power limits

Reactive power limits for the three FACTS devices are represented as:

(31) $$Q_{\text{TCSC}}^{\min}\leq Q_{\text{TCSC}}\leq Q_{\text{TCSC}}^{\max}$$

(32) $$Q_{\text{STATCOM}}^{\min}\leq Q_{\text{STATCOM}}\leq Q_{\text{STATCOM}}^{\max}$$

(33) $$Q_{\text{UPFC}}^{\min}\leq Q_{\text{UPFC}}\leq Q_{\text{UPFC}}^{\max}$$

Where $Q_{\text{TCSC}}^{\min}$, $Q_{\text{STATCOM}}^{\min}$ , $Q_{\text{UPFC}}^{\min}$ , $Q_{\text{TCSC}}^{\max}$ , $Q_{\text{STATCOM}}^{\max}$ , $Q_{\text{UPFC}}^{\max}$ represents minimum and maximum reactive power limits for the respective FACTS devices.

### 3.8 Thermal limits

The line thermal limit is expressed as:

(34) $$S_{\text{ij}}\leq S_{\text{ij}}^{\max}$$

Where $S_{i}^{\max}$ is the thermal limit for line i-j and $S_{\text{ij}}$ is the apparent power flowing in line i-j.

## 4 Methodology

FACTS devices have a huge potential of improving existing power systems for better performance. The promising results shown by FACTS devices when applied to improve system loadability, stability and security has attracted many researchers to investigate on the efficient FACTS optimization techniques. The optimization techniques for FACTS can be classified into three: analytical approaches, linear programming and Meta-Heuristic Optimization (MHO) methods [7]. Linear programming and analytic approaches are inefficient when solving constrained FACTS optimization problems. MHO techniques are robust and efficient in solving most of the modern-day power system problems [12]. The use of MHO techniques in solving non-linear and multimodal problems is preferred because these techniques are not restricted by differentiability and convexity of the objective functions. The search direction of MHO techniques is not dictated by initial solutions, a feature that guarantees global solution [38]. As a result, analytical and linear programming methods are being phased out and getting replaced by MHO methods. Thus, MHO algorithms are widely applied when solving the problem of optimal FACTS allocation. PSO and GA are well-known examples of MHO techniques that have been used for optimal sizing and placement of FACTS devices [1]. However, the original versions of these algorithms suffer from the problem of local stagnation. Hybrid algorithms are important because they help in solving specific problems that arise from standalone algorithms [39]. In this paper, the use of hybrid algorithm established harmony between diversification and intensification.

### 4.1 PSO overview

PSO is a useful swarm-intelligence algorithm that was introduced by Dr. Kennedy and Eberhart in 1995 [7]. This algorithm imitates the social behavior of flock or birds to find optimum solutions for optimization problems [8,40,41]. Under PSO, each particle moves within the search space in search for the best position for food, which symbolizes the optimal solution. Each particle has a position $X_{i}$, which represents a potential solution and this position is updated continuously as the particles moves at a velocity $V_{i}$ to better positions. By comparing the previous and current position, the particles can keep track of their best personal positions ($P_{best}$). Particles also compare their positions within themselves and the best position of the entire swarm is the best global position ($G_{best}$). Hence, PSO has both the cognitive and the social components; which allows for cooperation by the swarm members to reach high levels of intelligence. Acceleration coefficients $c_{1}$ and $c_{2}$ together with random numbers $r_{1}$ and $r_{2}$ are used to accelerate the personal and global experiences respectively. An inertia factor *w* multiplies the current movement of the particles. Equations (35), (36) represent the position and velocity of particles as they explore the search space.

(35) $$V_{i}^{t+1}=wV_{i}^{t}+r_{1}c_{1}\left(P_{\text{best},i}^{t}-X_{i}^{t}\right)+r_{2}c_{2}\left(G_{\text{best},i}^{t}-X_{i}^{t}\right)$$

(36) $$X_{i}^{t+1}=X_{i}^{t}+V_{i}^{t+1}$$

The inertia factor at iteration k is evaluated as:

(37) $$w=w_{\max}-\left(\frac{w_{\max}-w_{\min}}{\text{maximum}\;\text{iterations}}\right).k$$

PSO is attractive when solving the highly-constrained FACTS optimization problem because of its simplicity and easy implementation since it has few parameters for tuning [7]. However, the traditional PSO suffers a risk of getting trapped on local optima which can lead to premature convergence [36]. The standard PSO lacks a mechanism that can move particles from local optima. Thus, it becomes hard for the particles to escape local optima once the swarm has converged at the local positions. In this paper, the main aim to hybridize IPSO with GA is to introduce a mechanism for escaping local optima through the introduction of mutation operator from GA.

### 4.2 IPSO

The performance of PSO when optimizing FACTS devices can be affected by parameter setting. The three major parameters that can affect the performance of PSO are; *w,* $c_{1}$ and $c_{2}$. Using high values of the inertia weight causes erratic movement of the particles while low values of this parameter can result in stagnation of the particles [42]. When a high value of the cognitive acceleration coefficient $c_{1}$ is used, exploration is weakened. Again, high values of the social acceleration coefficient $c_{2}$ weakens the exploitation capability of the particles. In most cases for the traditional PSO, $c_{1}=c_{2}=2$ and $w_{\min}=0.4$ while $w_{\max}=0.9$ [42]. Proper tuning of these parameters is essential to improve the computational efficiency of PSO. For IPSO, constriction coefficient can limit the velocity equation to ensure gradual convergence of particles towards the best global solution. The inclusion of constriction coefficient helps to avoid the particles from overshooting leading to a stable convergence. Furthermore, the use constriction coefficient reduces computational time and burden by restricting the particles from moving too far away from the global solution. The constriction coefficient (χ) is expressed as:

(38) $$\chi =\frac{2}{|2-\emptyset -\sqrt{\emptyset^{2}-4\emptyset }|}$$

(39) $$\emptyset =c_{1}+c_{2}$$

The new equation for updating the particle's velocity becomes:

(40) $$V_{i}^{t+1}=\chi .\left[wV_{i}^{t}+r_{1}c_{1}\left(P_{\text{best},i}^{t}-X_{i}^{t}\right)+r_{2}c_{2}\left(G_{\text{best},i}^{t}-X_{i}^{t}\right)\right]$$

### 4.3 GA overview

GA is an evolutionary based technique that is motivated by the Darwin theory on natural selection [36]. At the start, a random population is set that represents potential solution to the problem. The fitness of the individuals in the initial population is calculated where individuals with good fitness survives. As the algorithm evolves, new possible solutions closer to the best solution referred to as offsprings are generated through selection, crossover and mutation genetic processes from the previous parents [43]. Although GA has been applied in many optimization problems, it suffers from the problem of local trapping and slow convergence rates. In GA, global optimum is missed especially in problems where individuals in the population are too similar. Hence, GA also suffers the problem of premature convergence when applied in its original form.

### 4.4 Proposed method: GA-IPSO

**Fig. 8:**
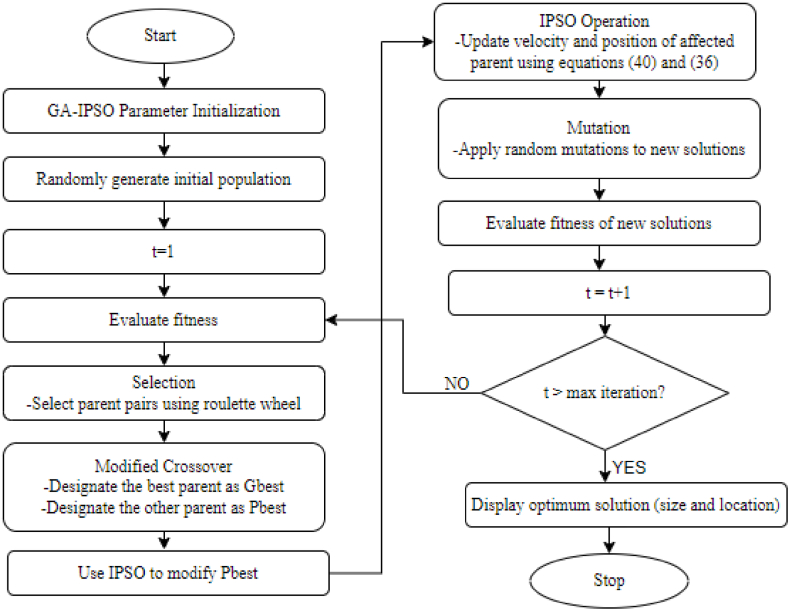
Proposed genetic algorithm-improved particle swarm optimization (GA-IPSO).

In this paper, a hybrid of GA and IPSO is used where the strengths of the algorithms are combined for better results. Metaheuristic algorithms should establish a good balance between exploitation and exploration so as to achieve good performance [44]. From the above discussions, the original forms of GA and PSO suffer from a common problem of local trapping. Hybridization of GA and IPSO creates a good balance between exploitation and exploration, solving the problem of premature convergence on the parent algorithms. The GA creates diversity in the proposed approach because it makes use of different evolutionary modes like crossover and mutation, which reduces the risk of falling to local optimum. The mutation operator in the GA provides a mechanism to escape from the local optima, which is lacking in the standard PSO. In this GA-IPSO approach, initial population is set and the fitness of all the individuals present in the population is evaluated. Each solution in the initial population represents a certain size of FACTS device and location. The roulette wheel selection approach is applied for the selection of the best two ranking parents based on fitness function [45]. Each parent represents a particular FACTS size and location on the test power network. Under the conventional GA, crossover between the two parents is performed where sharing of chromosomes between the two current parents produces two off-springs to act as the new parents in the next generation. In the proposed GA-IPSO, instead of the traditional crossover; the fitness of the two current parents is evaluated to designate one parent as ($G_{\text{best}}$) and the other parent as ($P_{\text{best}}$). Using the IPSO process, the velocity and position of one parent is updated while the other parent's position ($G_{\text{best}}$) remains unchanged. To introduce diversity, mutation is applied to the new solution before fitness is evaluated again. Each new solution represents a particular FACTS size and location. The modified crossover through the hybridization approach increases the possibility of a global optimal solution, hence overcoming the problem of getting trapped at the local optimum. The inclusion of IPSO in the crossover operation of GA reduces the complexity of this step. Again, this helps in reducing the overall computation time of GA-IPSO. Therefore, the GA-IPSO algorithm yields better results as compared to other meta-heuristic algorithms. This is because GA offers strong exploration through selection, crossover and mutation while IPSO focuses on exploitation. Thus, hybridization of these two algorithms offers good balance between exploitation and exploration of the search space. In addition, the use of constriction coefficient in IPSO controls the velocity of the particles, which prevents overshooting while promoting fast and stable convergence. The flow chart for the GA-IPSO approach used in the paper is represented in Fig. 8.

## 5 Results and discussion

**Fig. 9:**
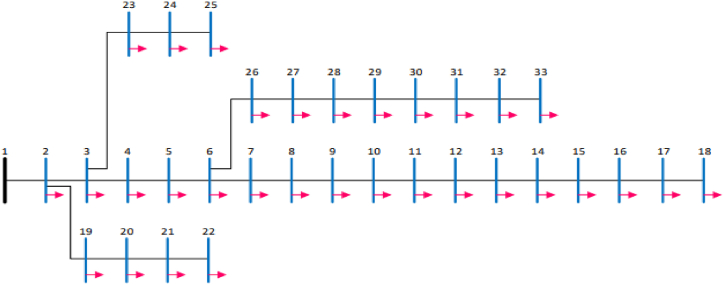
Ieee 33 bus system [46].

The IEEE 33 Bus System is a RDS that was used to test the effectiveness of the GA-IPSO method in optimal sizing and placement of FACTS devices. The bus and line data for the IEEE 33 bus system was extracted from Ref. [46]. The proposed method was programmed and executed using MATLAB software. Fig. 9 shows the bus system used in this paper.

### 5.1 Selection of weight factors

A multi-objective function involving minimization of PL, VD and OC was considered in this paper. The GA-IPSO algorithm was applied to the objective function expressed in equation (27). To select the most appropriate weights for the three objective functions, installation of the STATCOM was used as the case study. The algorithm was executed at different weighting factors as recorded in Table 3.

Table 2 is a summary of the GA-IPSO algorithm parameters used in this study:

**Table 2:** Genetic Algorithm-Improved Particle Swarm Optimization (GA-IPSO) parameters.

Parameter	Meaning	Symbol	Value
Population Size	Candidate Solutions	N	10
Iterations	Repeated process for improving solution	k	50
Crossover probability	Combination of two parent solutions during an iteration.	$p_{c}$	0.8
Mutation probability	Likelihood of a candidate solution altering during an iteration.	$p_{m}$	0.09
Cognitive constant	Tendency of a particle to move towards personal best position	$c_{1}$	2.2
Social constant	Tendency of a particle to move towards global best position	$c_{2}$	2.2
Inertia weight	Limits within which the inertia weight can vary.	$w_{\min}$, $w_{\max}$	−10, 10

**Table 3:** Different weight factors for multi-objective function considering STATCOM installation.

Weighting Factors	Bus	Size (Mvar)	FACTS Cost ($/Kvar)	Power Losses (MW)	Minimum Voltage (p.u)
(0.7,0.2,0.1)	29	52.96	123.25	178.706	0.9166 @bus18
(0.8, 0.1,0.1)	29	41.06	125.78	194.51	0.9142 @bus 18
(0.1,0.8,0.1)	29	92.28	124.90	189.61	0.9147 @bus 18
(0.1,0.1,0.8)	29	46.08	125.70	194.08	0.9142 @bus18
(0.2,0.7,0.1)	29	46.34	125.59	193.49	0.9143 @bus 18
(0.2,0.1,0.7)	29	123.04	124.56	187.63	0.9150@ bus 18
(0.6,0.3,0.1)	29	61.76	124.85	189.20	0.9149 @bus 18
(0.3,0.6,0.1)	29	46.32	125.46	192.78	0.9144 @bus18
(0.1,0.3,0.6)	29	73.97	125.26	191.68	0.9145 @bus18
(0.2,0.6,0.2)	29	52.88	124.66	188.03	0.9151 @bus 18
(0.6,0.2,0.2)	29	184.93	121.90	168.18	0.9180 @bus 18
(0.2,0.2,0.6)	29	92.68	124.10	184.66	0.9155 @bus18
(0.5,0.1,0.4)	29	184.93	122.61	174.23	0.9169 @bus 18
(0.5,0.4,0.1)	29	92.38	123.64	181.56	0.9160 @bus 18
(0.1,0.5,0.4)	29	52.94	125.49	192.92	0.9144 @bus 18

Among the different cases selected randomly, the most applicable weights emerged to be $\omega_{1}=$ 0.8, $\omega_{2}=$ 0.1 and $\omega_{3}=$ 0.1. These specific weights provided the best balance among the three objective functions under consideration by providing the lowest capacity of the STATCOM and improving the voltage magnitude at the weakest bus with the largest margin compared to the other cases in Table 3. Since power loss is the biggest problem in power networks, the obtained weights are appropriate because power loss is given the highest priority in the optimization problem. The optimization was carried out carried using the selected wights ($\omega_{1}=$ 0.8, $\omega_{2}=$ 0.1 and $\omega_{3}=$ 0.1) for the selected study cases.

Case 1 Separate Installation of FACTS devices.

Case 2 Combined Installation of FACTS devices.

### 5.2 Case 1: separate installation of FACTS devices

#### 5.2.1 Installation of TCSC only

For individual installation of TCSC, the proposed method (GA-IPSO) was executed at the most appropriate weights ($\omega_{1}=$ 0.8, $\omega_{2}=$ 0.1 and $\omega_{3}=$ 0.1). Prior to optimization, the active power losses for the test system were 202.68 kW. The cost originating from the energy losses can be evaluated using Equation (22), which gives an annual operating cost of $ ${1.59\times 10}^{5}$. For the base case, the minimum voltage magnitude was 0.9131 p.u at bus 18. Optimization of TCSC through GA-IPSO placed the device between bus 28 and 29 and reduced the active power losses of the bus system to 195.68 kW as represented in Table 4. The optimization of the TCSC on the test system resulted in 3.58 % power loss reduction. The voltage magnitude at bus 18 was improved to 0.9139 p.u. The optimum size for the TCSC was found to be 73.78 MVAr which falls within the allowable power limits. This is similar to the other FACTS devices discussed in the subsequent sections showing non-violation of power constraints by the proposed algorithm. The proposed method resulted in an investment cost for the TCSC of $150.30/KVar for a 73.78 MVAr TCSC. With the TCSC connected, the minimized operating cost of the power system was reduced to $ ${1.54\times 10}^{5}$ which is less as compared to the operating cost at the base case.

**Table 4:** Optimal solution for separate FACTS optimization with best fit weighting factors (0.8, 0.1, 0.1).

Parameters	Base Case	TCSC Only	STATCOM Only	UPFC Only
PL (kW)	202.68	195.68	194.51	146.40
MV @bus (p.u)	0.9131@18	0.9139@18	0.9142@18	0.9216@18
OC ($)	${1.59\times 10}^{5}$	${1.54\times 10}^{5}$	${1.53\times 10}^{5}$.	${1.15\times 10}^{5}$
OL (Bus)	–	28–29	29	29
OS (MVAr)	–	73.78	41.06	252.47

Key: PL-Power Losses, MV-Minimum Voltage, OC-Operating Cost, OL-Optimal Location, OS-Optimal Size.

#### 5.2.2 Installation of STATCOM only

The minimization of the multi-objective function by GA-IPSO positioned the STATCOM at the 29th bus of the IEEE-33 bus system. The optimum size for the STATCOM was 41.06 MVAr and the minimum investment cost was $125.78/KVAr. Optimization of STATCOM reduced power losses of the system from 202.68 kW to 194.5 kW. This is equivalent to 4.21 % active power loss reduction. The minimized cost of the system was $ ${1.53\times 10}^{5}$ which is lower than the operating cost of the base system. STATCOM improved the voltage magnitude at the bus with the lowest voltage from 0.9131 p.u to 0.9142 p.u.

#### 5.2.3 Installation of UPFC only

The GA-IPSO was applied for optimization of UPFC with the aim of reducing PL, VD and OC. The proposed method positioned UPFC at the 29th bus and improved the performance of the test network. The optimum size for the UPFC was 252.47 MVAr and the minimized investment cost of the device was 187.63 KVAr. The system power losses were reduced from 202.68 kW to 146.40 kW. This results in 38.44 % reduction of active power losses. Furthermore, the installation of UPFC improved the minimum voltage at bus 18 from 0.9131 p.u to 0.9216 p.u. Optimal allocation of UPFC through GA-IPSO reduced the system operating cost from $\${1.59\times 10}^{5}$ to $ ${1.15\times 10}^{5}$ as represented in Table 4.

**Fig. 10:**
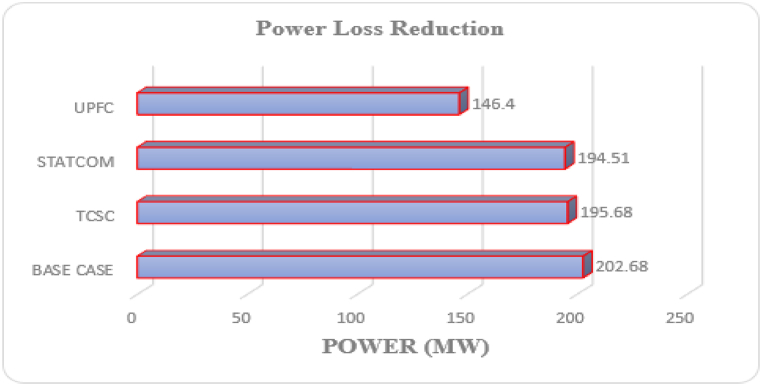
System power loss with different FACTS

The installation of individual FACTS devices on the test system produced varying results. Reduction of system power losses is one of the major reasons of using FACTS devices in power networks. Fig. 10 compares how the installation of different FACTS devices reduced real power losses. UPFC reduced the system losses by the largest value followed by STATCOM and then TCSC. UPFC produced better results than the other two FACTS devices in terms of loss reduction because it can control the three power transfer parameters.

**Fig. 11:**
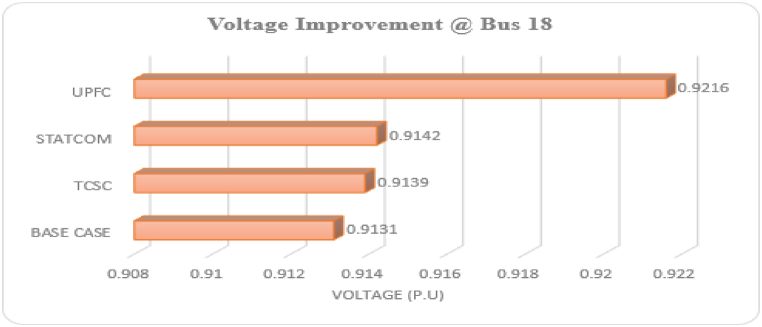
Voltage magnitude at bus 18 with different FACTS

Prior to the optimization of FACTS devices using GA-IPSO algorithm, the minimum voltage of the IEEE-33 bus system was at bus 18. Fig. 11 compares how the installation of different FACTS devices improved the voltage magnitude of this bus with respect to the base case scenario. UPFC produced the best results by improving the voltage magnitude at bus 18 with the largest margin. STATCOM and TCSC almost produced similar results with STATCOM slightly ahead of TCSC as illustrated in Fig. 11.

**Fig. 12:**
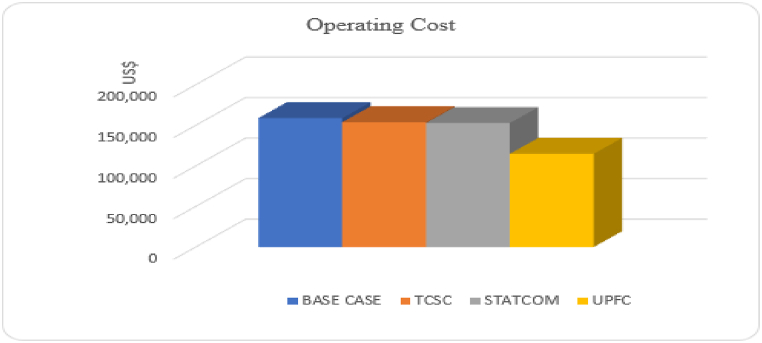
System operating cost with different FACTS devices.

The investment cost of FACTS devices is usually high, hence the need to evaluate their economic feasibility. For the base system operating without FACTS devices, the OC involves the cost resulting from energy losses. When FACTS devices are incorporated, the OC consists of both the energy losses costs and the FACTS investment cost. Fig. 12 compares the OC of the base system with the system operating under different FACTS devices. As shown, the installation of the three FACTS devices were all economically feasible because the OC was reduced across all the three cases. Again, UPFC reduced the operating cost with the largest value. Since UPFC reduced system losses by the biggest margin, this led to reduction of the cost of energy losses significantly.

**Fig. 13:**
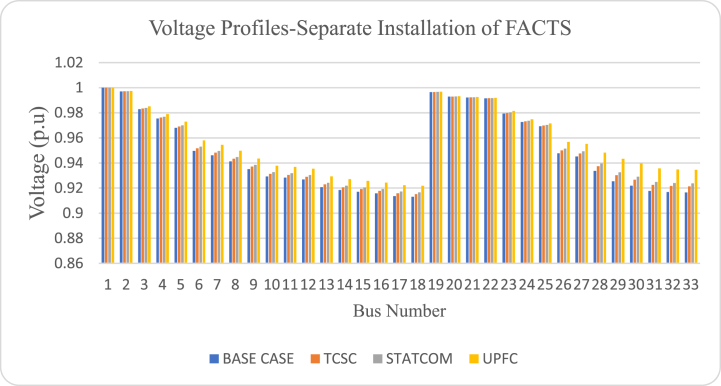
Voltage profiles for separate optimization of FACTS.

Fig. 13 illustrates the voltage profiles of the base case and improved voltage profiles after individual optimization of TCSC, STATCOM and UPFC. Individual optimization of the three FACTS devices using GA-IPSO approach improved the voltage profile of test system for all the three cases. Optimization of UPFC produced the best voltage profile as shown in Fig. 13. This illustrates the suitability of using UPFC in improving the voltage profile in power networks suffering from voltage related problems. Furthermore, according to Fig. 13, all voltage values are above 0.9 p.u and below 1.05 p.u. Based on equation (37), this shows that the voltage limit constraint is not violated by the proposed hybrid method.

### 5.3 Case 2: combined installation of FACTS devices

Using the GA-IPSO approach, the sizes and locations of different FACTS combinations were optimized while minimizing the PL, VD and OC. The four cases considered under combined optimization of FACTS devices include: optimization of TCSC and UPFC, optimization of UPFC and STATCOM, optimization of STATCOM and TCSC, optimization of STATCOM, TCSC and UPFC. Table 5 summarizes the optimal results obtained after combined optimization of the four different FACTS combinations.

**Table 5:** Optimal solution for combined FACTS optimization with best fit weighting factors (0.8, 0.1, 0.1).

Parameters	Base Case	TCSC + UPFC	UPFC + STATCOM	STATCOM + TCSC	STATCOM + TCSC + UPFC
PL (kW)	202.68	154.52	141.91	196.65	129.85
MV@bus(p.u)	0.9131@18	0.9202@18	0.9222@18	0.9137@18	0.9240@18
OC ($)	${1.59\times 10}^{5}$	${1.22\times 10}^{5}$	${1.12\times 10}^{5}$	${1.55\times 10}^{5}$	${1.03\times 10}^{5}$
OL(Bus)	–	28-29,24	29,24	29,23-24	23,28–29,24
OS(MVAr)	–	20.49,684.92	74.03,651.39	151.04,126.28	233.54,721.39,46.32

Key: PL-Power Losses, MV-Minimum Voltage, OC-Operating Cost, OL-Optimal Location, OS-Optimal Size.

Combined optimization of TCSC and UPFC reduced the power losses from 202.68 kW to 154.52 kW. This is equivalent to 31.17 % active power loss reduction. A combination of UPFC and STATCOM reduced active power losses by 42.82 % while combined optimization of STATCOM and TCSC minimized active power losses by 3.07 %. The highest power loss reduction was achieved under Combined optimization of STATCOM, TCSC and UPFC where active power losses were reduced by 56.09 %.

**Fig. 14:**
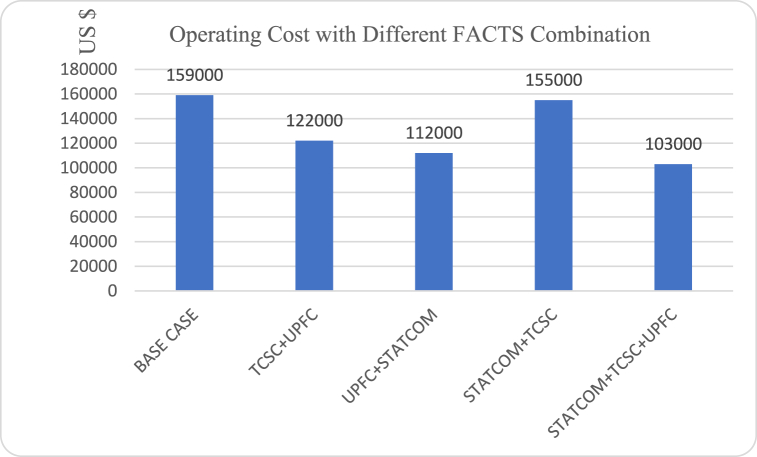
Operating cost with different FACTS combinations.

In all the four different FACTS combinations, the system operating cost was reduced to a lower value as compared to the base case system as shown in Fig. 14. Although installing two or three different types of FACTS in a power network results in higher investment cost, the combination improves the system performance to higher levels as compared to the installation of a single device. Improved performance through lower system losses and better voltage profile lowers the cost of running the network, which offsets the high installation costs. As shown in Fig. 14, the combinations that involves UPFC produced better results by reducing operating costs to lower values. The combination involving Combined installation of STATCOM and TCSC reduced the operating cost by a small margin. This is because under this combination, STATCOM controls the voltage magnitude while TCSC controls the line reactance. This leaves the phase angle uncontrolled, hence this combination cannot improve the power transfer capability of the system to the levels achieved in configurations where UPFC that controls all the three parameters of the power system is involved. A combination involving UPFC, STATCOM and TCSC proofed to be most effective in reducing the system's operating cost as shown in Fig. 14.

At the optimum solution, combination of the three FACTS devices reduced the operating cost to $ ${1.03\times 10}^{5}$. This combination brings together FACTS from all the three classes, which boosts the performance of the power system to desirable levels. Although installation of three different FACTS devices incurs high investment costs, this study shows that the benefits outweigh the investment costs if proper optimization of the FACTS sizes and position is done.

### 5.4 Comparison between separate and combined installation of FACTS devices

**Fig. 15:**
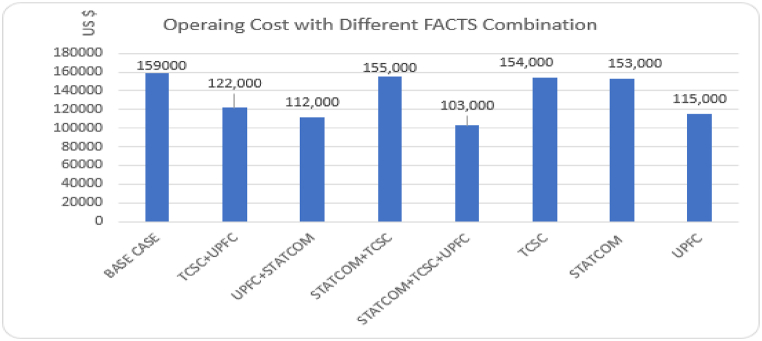
Operating cost for separate and combined installation of FACTS devices.

In terms of system operating cost, the different FACTS considered in this paper can be ranked as: STATCOM + TCSC + UPFC > UPFC + STATCOM > UPFC > TCSC + UPFC > STATCOM > TCSC > STATCOM + TCSC. The operating cost for all the different FACTS configurations is illustrated in Fig. 15. More economic benefits can be achieved if the three FACTS devices are combined and used in the same power network. Since each FACTS device has unique attributes, optimal combination of the FACTS leads to a high performing power network. Use of a single UPFC is more economically feasible on IEEE-33 bus network as compared to a combination of TCSC + UPFC and STATCOM + UPFC.

**Fig. 16:**
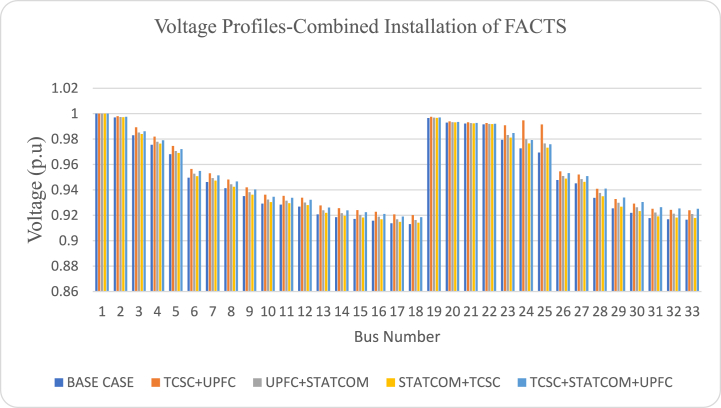
Voltage profiles for combined optimization of FACTS.

Fig. 16 compares the voltage profiles of the system under the different FACTS configuration for combined optimization case. The voltage profiles obtained after using combined FACTS are better as compared to the voltage profile of the base system. However, the best voltage profile is obtained when STATCOM, TCSC and UPFC are jointly optimized together and installed in the test power network as shown in Fig. 16.

### 5.5 Comparison of GA-IPSO approach with GA and IPSO

**Fig. 17:**
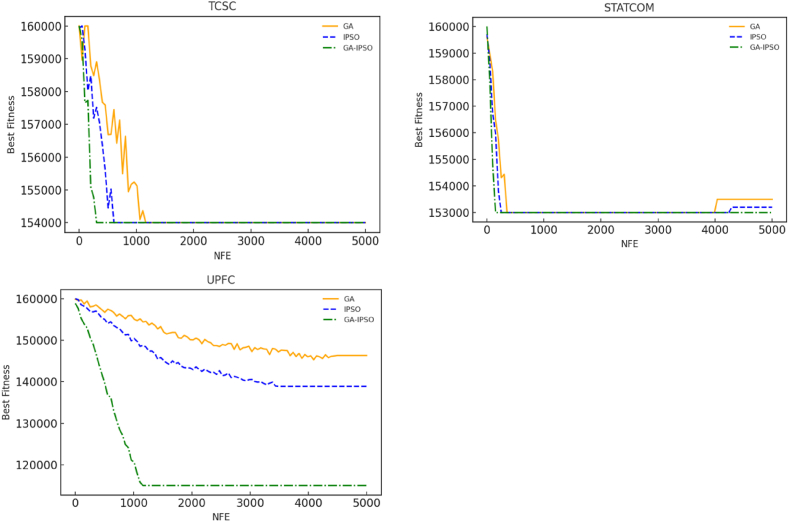
Convergence curves for separate installation of FACTS devices (Case 1).

**Fig. 18:**
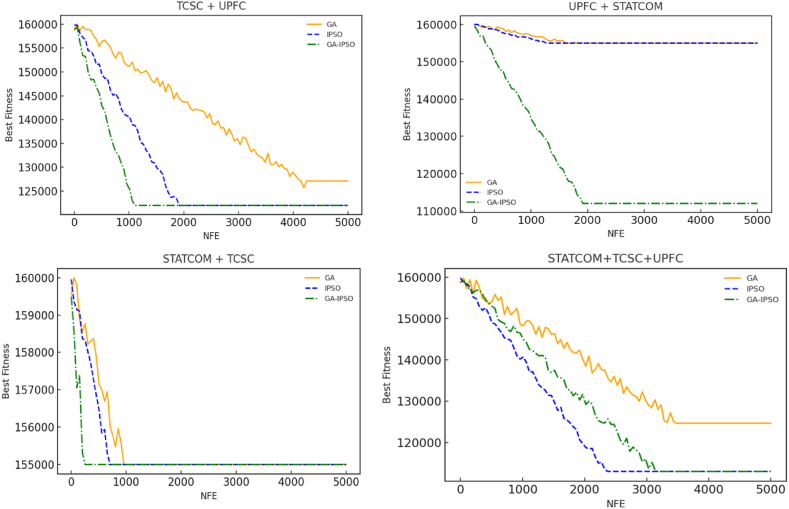
Convergence curves for combined installation of FACTS devices (Case 2).

To show the superiority of GA-IPSO algorithm, convergence curves for all the seven cases considered in this paper were compared under identical number of fitness evaluations (NFE). The convergence curves for the three cases considered under separate installation of FACTS are shown in Fig. 17. Under combined installation of FACTS, the convergence curves for the four cases considered are illustrated in Fig. 18. The initial population for all the algorithms is set at the same value at the start to achieve a fair comparison. Under separate installation of FACTS, GA-IPSO found the global optimum faster than GA and IPSO for all the three cases as shown in Fig. 17. This trend repeated itself under the combined installation of FACTS devices with an exception of only case (STATCOM + TCSC + UPFC) as shown in Fig. 18. Thus, judging from the convergence curves, GA-IPSO converges faster than GA and IPSO when solving the problem of FACTS optimization. The superior convergence behavior of GA-IPSO can be attributed to hybridization that balances exploitation and exploration. Hybridization also reduces time complexity of GA-IPSO by simplification of the crossover process as explained in section 4.4. Hence, apart from improving the accuracy of the solution, GA-IPSO reduces the computation time when used in FACTS optimization problem.

The performance of GA-IPSO was compared with that of GA and IPSO as delineated in Table 6 and Table 7. In Table 6, GA-IPSO achieved the highest Power Loss Reduction (PLR) for all the three cases considered under separate installation of FACTS devices. Similarly, GA-IPSO reduced power losses by the highest percentage except for one case (STATCOM + TCSC) under the combined installation of FACTS as shown in Table 7. Overall, GA-IPSO out performed GA and IPSO for the six out of the seven cases considered in this paper. This shows prolific convergence traits of GA-IPSO algorithm when solving the problem of FACTS optimization. Again, Table 6, Table 7 shows that GA-IPSO converges faster than GA and IPSO in the cases considered except for one case (STATCOM + TCSC + UPFC). Fast convergence of GA-IPSO is attributed to the reduced computational burden of the proposed algorithms.

**Table 6:** Comparative results between GA, IPSO and GA-IPSO for separate installation of FACTS.

FACTS	Measure	GA	IPSO	GA-IPSO(Proposed)
TCSC	PLR (%)	3.20	2.18	3.58
	Time (s)	3.21	2.96	2.58
STATCOM	PLR (%)	3.52	3.52	4.21
	Time (s)	2.74	2.70	2.67
UPFC	PLR (%)	23.11	29.35	38.44
	Time (s)	4.85	4.17	3.54

Key: PLR-Power Loss Reduction.

**Table 7:** Comparative results between GA, IPSO and GA-IPSO for combined installation of FACTS.

FACTS	Measure	GA	IPSO	GA-IPSO(Proposed)
TCSC + UPFC	PLR (%)	30.56	30.24	31.17
	Time (s)	10.23	6.17	3.22
UPFC + STATCOM	PLR (%)	40.23	42.12	42.82
	Time (s)	7.97	7.92	6.51
STATCOM + TCSC	PLR (%)	3.12	4.50	3.07
	Time (s)	3.84	3.11	2.54
STATCOM + TCSC + UPFC	PLR (%)	51.17	52.32	56.09
	Time (s)	8.89	6.77	7.44

Key: PLR-Power Loss Reduction.

### 5.6 Comparison of GA-IPSO approach for FACTS optimization with other works

The use of GA-IPSO was effective in solving the FACTS optimization problem. In Ref. [2], PSO algorithm was used for combined optimization of TCSC and UPFC and active power losses were reduced by 20.60 %. In this work, optimal allocation of TCSC and UPFC by GA-IPSO algorithm reduced the system's active power losses by 31.17 %, which shows the effectiveness of the hybrid optimization approach. Use of GA in Ref. [47] to optimize UPFC reduced the system losses by 1.25 %. Optimization of UPFC in IEEE 33 bus system using Differential Evolution Algorithm (DEA) reduced the system losses by 0.015 % in Ref. [48]. In this paper, installation of single UPFC using the GA-IPSO technique reduced the losses of the test power system by 38.44 %. When compared to Refs. [2,5,17,48] where Whale Optimization Algorithm (WOA), Autonomous Group Particle Swarm Optimization-Grey Wolf Optimization (AGPSO-GWO), Adaptive Particle Swarm Optimization (APSO) and Improved Grey Wolf Optimization (IGWO) were used respectively to optimize FACTS size and location, the proposed GA-IPSO approach produced better results as shown in Table 8.

**Table 8:** Comparison of GA-IPSO with different optimization algorithms.

FACTS	Algorithm	Power Loss Reduction	FACTS	Algorithm	Power Loss Reduction
TCSC	IGWO [49]	3.35 %	STATCOM	APSO [17]	2.87 %
	AGPSO-GWO [5]	3.34		GA-IPSO	4.21 %
	GA-IPSO	3.58 %	UPFC	GA [46]	1.25 %
TCSC + UPFC	WOA [2]	21.18 %		DEA [47]	0.015 %
	PSO [2]	20.60 %		IGWO [49]	13.66 %
	GA-IPSO	31.17 %		GA-IPSO	38.44 %

## 6 Conclusion

Different Meta-Heuristic Optimization (MHO) algorithms have been applied extensively to solve the FACTS optimization problem. This paper focused on optimization of size and location for Static Synchronous Compensator (STATCOM), Thyristor Controlled Series Capacitor (TCSC) and Unified Power Flow Controller (UPFC). Particle Swarm Optimization (PSO) and Genetic Algorithm (GA) are popular algorithms used in optimization of FACTS devices. The standard forms of these algorithms suffer from the problem of local trapping. For improved convergence characteristics and efficiency, a hybrid approach (GA-IPSO) has been suggested in this paper. The GA-IPSO hybrid approach was applied to optimize FACTS devices on IEEE-33 Bus System. TCSC, STATCOM and UPFC were optimized both individually and in a combined manner. The FACTS devices were optimized to minimize power losses, voltage deviation and operating cost. The weights for the multi-objective function used in this paper were $\omega_{1}=$ 0.8 for PL, $\omega_{2}=$ 0.1 for VD and $\omega_{3}=$ 0.1 for OC. These weight factors provided the best balance of the objective function with the highest significance given to power losses. Under separate allocation of FACTS devices, UPFC produced the best results. Under Combined optimization of FACTS devices, a combination of STATCOM, TCSC and UPFC emerged as the best combination that offers minimum power losses and minimum operating costs. When compared to other algorithms such as PSO, GA and DEA, the proposed hybrid technique produced better results at a good convergence rate. Since the model developed in this work allows for optimization of different FACTS configurations, the paper findings can assist power system planners in examining and selecting the most appropriate FACTS that fits their power networks. One challenge of the proposed GA-IPSO is that it has many tuning parameters because it combines the parameters of the individual GA and IPSO. Thus, applying the proposed method in a highly dimensional problem is worth of attention. In future, the authors intend to apply and evaluate the proposed GA-IPSO algorithm on practical and benchmark functions with different dimensions.

## CRediT authorship contribution statement

**Urbanus Mwanzia Ngei:** Writing – original draft, Software, Methodology, Investigation, Data curation, Conceptualization. **Abraham Mutunga Nyete:** Supervision, Methodology, Conceptualization. **Peter Musau Moses:** Writing – review & editing, Supervision, Methodology, Conceptualization. **Cyrus Wekesa:** Writing – review & editing, Supervision, Methodology, Conceptualization.

## Declaration of competing interest

The authors declare that they have no known competing financial interests or personal relationships that could have appeared to influence the work reported in this paper.
